# Short-term postoperative changes in the choroidal vascularity index in patients with a unilateral epiretinal membrane

**DOI:** 10.1186/s12886-022-02748-6

**Published:** 2023-02-13

**Authors:** Kaiming Ruan, Yun Zhang, Dan Cheng, Yilin Qiao, Yufeng Yu, Minhui Wu, Xueying Zhu, Jiwei Tao, Meixiao Shen, Lijun Shen

**Affiliations:** 1 The Affiliated Eye Hospital of Wenzhou Medical University, 618 Fengqi East Road, Zhejiang, 310000 Hangzhou China; 2 Department of Ophthalmology, Zhejiang Provincial People’s Hospital, Hanghzhou, Zhejiang, China

**Keywords:** Choroid, Optical coherence tomography, Choroidal vascular index, Idiopathic epiretinal membrane, Vitrectomy

## Abstract

**Background:**

To investigate short-term choroidal structural and vascular changes after epiretinal membrane (ERM) surgery.

**Methods:**

In this retrospective study, 65 patients with unilateral ERM underwent pars plana vitrectomy combined with cataract surgery and were examined one day before surgery and one week, one month, and three months after surgery. Choroidal thickness (CT) and choroidal vascular index (CVI) were evaluated using horizontal enhanced depth imaging optical coherence tomography (EDI-OCT) scans and were further calculated using semi-automatic algorithms using MATLAB R2017a.

**Results:**

Preoperatively, CVI was higher in eyes with ERM (61.70 ± 5.17%) than in fellow eyes (59.99 ± 5.26%). CVI increased significantly at one week after surgery (62.14 ± 5.02%) and decreased at 1 and 3 months after surgery (60.76 ± 4.97% and 60.4 ± 4.83%, respectively). The change was pronounced in the nasal region (*p* < 0.001) and central region (*p* < 0.05). CT in the temporal macula increased at 1 week (239.65 ± 72.98 μm) after surgery and decreased at 1 and 3 months after surgery (222.15 ± 71.91 μm and 222.33 ± 65.72 μm, respectively; *p* < 0.01).

**Conclusions:**

Short-term postoperative variations in the choroid have been demonstrated in eyes with ERM. This may be related to the release of macular traction. CVI assessment using EDI-OCT may be a useful tool for investigating choroidal structural changes accompanying ERM and postoperative period.

## Background

The idiopathic epiretinal membrane (iERM) is a fibrocellular growth at the vitreoretinal interface that can constrict and alter the structure of the retina, and its incidence increases with age [1, 2]. These changes can lead to vision loss, metamorphopsia, and aniseikonia [3, 4]. When indicated, ophthalmologists surgically remove the membrane and relieve the traction on the retina [5].

Previous studies have demonstrated that the choroid and retina, are affected in iERM, and that choroidal thickness decreased 3 months after surgery [6]. More recently, the choroidal vascularity index (CVI) was considered a new and more reliable metric for characterizing choroidal vascularity, which was computed by dividing the luminal area (LA) by the total subfoveal circumscribed choroidal area (TCA) based on enhanced depth imaging (EDI) optical coherence tomography (OCT) [7, 8]. Compared to CT, which is associated with multiple patient factors including age, axial length, intraocular pressure, and systolic blood pressure, CVI is a more robust and resistant choroidal parameter [8].

Short-term complications following surgery have been widely reported in patients with ERM [9, 10]. Datlinger et al. reported postoperative movement of the fovea one day after successful surgery for iERM [11]. Watanabe et al. reported that metamorphopsia and retinal displacement decreased significantly 3 months after epiretinal membrane surgery [12]. In addition, early postoperative variations in intraocular pressure, hypotony, choroidal detachment, and corneal decompensation have been reported. However, knowledge about the short-term changes in CVI after surgery in patients with ERM is limited. Furthermore, few studies have investigated changes in CVI in different regions in patients with ERM.

This study aimed to evaluate short-term postoperative changes in CT and CVI in eyes with ERM using EDI-OCT. In addition, we compared the differences in choroidal parameters between the affected and fellow eyes.

## Methods

### Participants

The study protocol adhered to the tenets of the Declaration of Helsinki and was approved by the Ethics Committee of Wenzhou Medical University. All patients have been informed of the purpose of the experiment and signed informed consent. Patients with ERMs were recruited from January to December 2021 at the Affiliated Eye Hospital of Wenzhou Medical University in Hangzhou.

Each patient underwent a complete ophthalmic examination, including dilated fundus examination by two experienced specialists (Shen Lijun and Cheng Dan), best-corrected visual acuity (BCVA) measurement using Snellen charts, intraocular pressure measurement (Goldmann applanation tonometry), and spectral-domain OCT (SD-OCT, Heidelberg Engineering, Heidelberg, Germany).

The inclusion criteria were as follows: patients with clinically diagnosed unilateral ERM who underwent surgical removal, with a minimum follow-up of three months. The exclusion criteria were as follows: eyes with other intraocular diseases such as uveitis, age-related macular degeneration, diabetic retinopathy, severe cataract, high myopia (> − 6 diopters), glaucoma, vitreomacular traction syndrome; eyes with previous vitreoretinal surgery; and OCT images of poor quality. No obvious postoperative complications occurred after surgery, such as, Irvine-Gass syndrome，macular hole, retinal detachment, and endophthalmitis. Moreover, considering the impact of complex surgery on the choroid, we excluded intraoperative retinal photocoagulation for retinal tears, holes or rhegmatogenous retinal detachments.

### EDI OCT image acquisition and analysis

Patients and controls underwent a horizontal single-line EDI-OCT scan encompassing the fovea (Heidelberg Engineering, Heidelberg, Germany). EDI-OCT images were obtained between 9:00 AM and 12:00 AM to restrict the effect of the circadian rhythm on choroidal parameters [13]. The choroidal thickness (CT) and choroidal vascularity were assessed using the scan images. The choroid was defined as the area between the retinal pigment epithelium (RPE)–Bruch’s membrane complex and the choroid–sclera interface in SD-OCT images. After semiautomatic choroidal segmentation using a custom method built in MATLAB R2017a (MathWorks, Natick, MA, USA), a trained examiner (Ruan Kaiming) manually modified segments of the RPE–Bruch’s membrane complex and choroid–sclera interface. After segmentation, each image was binarized in MATLAB R2017a using custom-created algorithms to demarcate the LA and stromal area using Niblack’s auto local threshold, which was proposed by Sonoda et al. [14]. The mean macular CT, TCA, LA, and stromal area were estimated after image processing, and the size was adjusted to account for changes in magnification between the eyes due to differing ALs. The ratio of LA to TCA was used to calculate CVI. The region of interest was defined as a 6-mm macular region centered on the fovea.

**Fig. 1:**
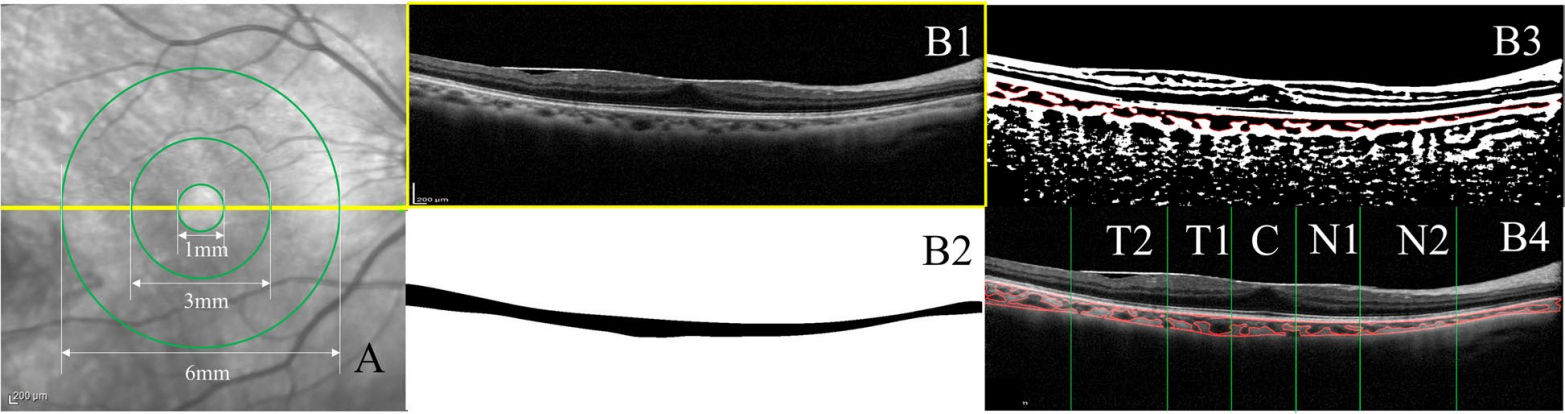
Choroidal vascularity index (CVI) and choroidal thickness were measured in Macular according to ETDRS grid (**A**) Showing steps (**B1-4**) using semi-automatic algorithms in MATLAB R2017a. T1, temporal parafovea; T2, temporal perifovea; C, center; N1, nasal parafovea; N2, nasal perifovea

The macular zone was split into three concentric rings with diameters of 1 (central fovea, C), 3 (parafovea), and 6 mm (perifovea). The central region centered on the fovea had a diameter of 1 mm. N1 and N2 nasal regions extended from an inner diameter of 1 mm and 3 mm to an outer diameter of 3 mm and 6 mm, respectively. T1 and T2 temporal regions extended from an inner diameter of 1 mm and 3 mm to an outer diameter of 3 mm and 6 mm, respectively. CT and CVI in each region were calculated using horizontal B-scans (Fig. 1).

In addition, according to Govetto et al., we categorized ERM into four stages using B-scan images across the macula [15]. Briefly, stage 1 is defined as the presence of the foveal pit and well-defined retinal layers; stage 2 is defined as the absence of the foveal pit and well-defined retinal layers; stage 3 is defined as the absence of the foveal pit and well-defined retinal layers, and the presence of ectopic inner foveal layers; stage 4 is defined as the absence of the foveal pit, presence of ectopic inner foveal layers, and disrupted retinal layers.

### Surgical procedure

All procedures were performed by a single surgeon (Shen, Lijun). All 65 epiretinal membrane eyes received retrobulbar anesthesia preoperatively. We dotted a mark on the sclera at a distance of 3.5 mm from the corneoscleral limbus with an angle ruler. The trocar was first inserted through the sclera with a 30-degree angle and then oriented tangentially to the sclera. The conjunctiva and sclera were penetrated with the trocar, and a microcannula was implanted. Standard 3-port pars plana vitrectomy (PPV) was performed using 23-gauge instruments (Constellation Vision System, Alcon Laboratories, Fort Worth, Texas, USA). Cataract surgery was performed in all 65 phakic eyes. A 0.025% indocyanine green solution (DANDONG YICHUANG PHARMACEUTICAL CO., LTD) was injected into the vitreous cavity and washed out no later than 3 s using a 23-gauge cutter. The ERM and inner limiting membrane was peeled by careful grasping with forceps (Grieshaber Revolution DSP, ILM Forceps, Alcon) without damaging the retina to approximately 4-disc diameters centered on the macula. In 65 epiretinal membrane eyes, complete posterior vitreous detachment (PVD) accounted for the majority of epiretinal membrane eyes (*n* = 52), and the remaining few (*n* = 13) were incomplete PVD and induced intraoperatively. All patients underwent fluid–air exchange, and the sclerotomies were sutured by 8-0 polypropylene. And there was no sign of leakage. No complications due to leakage such as low IOP occurred postoperative. Patients were instructed to remain in the prone position for 3–5 days postoperatively. Within one month after epiretinal membrane surgery, we routinely used levofloxacin eye drops 4 times a day and tobramycin-dexamethasone 4 times a day at first week and decreasing once a week. None of these drugs have been reported to have an effect on CVI. No medication for controlling IOP were used.

### Statistical analysis

Statistical analysis was performed using Statistical Package for the Social Sciences statistical software (SPSS Inc., Chicago, IL, United States). The mean with standard deviation for the normal distribution is shown in the tables. BCVA was converted to the logarithm of the minimum angle of resolution (logMAR) for data analysis. For logMAR of BCVA, paired t-tests were applied. To compare CVI and CT between eyes with ERM and fellow eyes, paired t-tests were used. To compare CVI and CT at 1 day before surgery with those at 7, 30, and 90 days after surgery, repeated-measures ANOVA tests were applied, and post hoc analyses were performed. Statistical significance was set at *p* < 0.05.

## Results

This study included 130 eyes of 65 patients with iERM. The mean age was 61.42 ± 11.30 years; 38 (58.5%) patients were male and 27 (41.5%) were female. Among eyes with ERM, there were 9, 31, and 25 in stages 2, 3, and 4, respectively. Eyes with ERM had worse visual acuity than the fellow eyes (logMAR, 0.688 ± 0.678 vs 0.155 ± 0.357, *p* < 0.001). Compared with the preoperative values, there was no significant difference in visual acuity at 1 week (logMAR, 0.653 ± 0.573, *p* = 0.928) after surgery in the ERM group. Visual acuity improved significantly at 1 month (logMAR, 0.416 ± 0.432, *p* < 0.01) and 3 months after surgery (0.346 ± 0.448, *p* < 0.01) (Table 1).

**Table 1:** Comparison of Clinical Characteristics Between Eyes with ERM and Fellow Eyes

	Fellow Eye	Eyes with ERM	*P* Value
Preoperative examination			
Number	65	65	–
Age, years	61.42 ± 11.30		–
Sex, male/female	38/27		–
Stage (0,1,2,3,4)		0,0,9,31,25	–
LogMAR, BCVA	0.155 ± 0.357	0.688 ± 0.678	< 0.001^a^
One week after surgery			
LogMAR, BCVA		0.653 ± 0.573	0.928^b^
One month after surgery			
LogMAR, BCVA		0.416 ± 0.432	< 0.01^b^
Three months after surgery			
LogMAR, BCVA		0.346 ± 0.448	< 0.01^b^

*ERM* epiretinal membrane, *LogMAR* logarithm of the minimal angle of resolution;

*BCVA* best corrected visual acuity

^a^ Significant difference between fellow eyes and eyes with ERM；

^b^ Significant difference between postoperative and preoperative examination

Compared to the fellow eyes, CVI was higher in the total macular (61.70 ± 5.17% vs 59.99 ± 5.26%, *p* < 0.01) and central regions (62.11 ± 6.84% vs 60.26 ± 6.52%, *p* < 0.01) in eyes with ERM (Table 2). CVI comparison with the fellow eye reduce interpersonal CVI difference, but an intrapersonal CVI difference still exists.

**Table 2:** Comparison of the Preoperative CVI and CT Between Eyes with ERM and Fellow Eyes

	Fellow Eyes	Eyes with ERM	*P* Value
CVI (%)			
Total	59.99 ± 5.26	61.70 ± 5.17	< 0.01
N2	58.89 ± 7.09	58.74 ± 7.65	0.871
N1	61.37 ± 5.95	62.51 ± 5.93	0.123
C	60.26 ± 6.52	62.11 ± 6.84	< 0.05
T1	60.76 ± 6.48	62.32 ± 7.71	0.172
T2	60.01 ± 7.71	62.24 ± 7.97	0.069
CT (μm)			
Total	204.57 ± 69.71	209.80 ± 63.26	0.440
N2	171.74 ± 66.35	176.61 ± 71.40	0.517
N1	209.32 ± 77.50	206.12 ± 72.48	0.668
C	216.57 ± 82.85	223.91 ± 78.20	0.398
T1	222.10 ± 78.60	235.47 ± 75.75	0.122
T2	214.56 ± 72.83	218.91 ± 63.11	0.601

*ERM* epiretinal membrane, *CVI* choroidal vascularity index, *CT* choroidal thickness

**Fig. 2:**
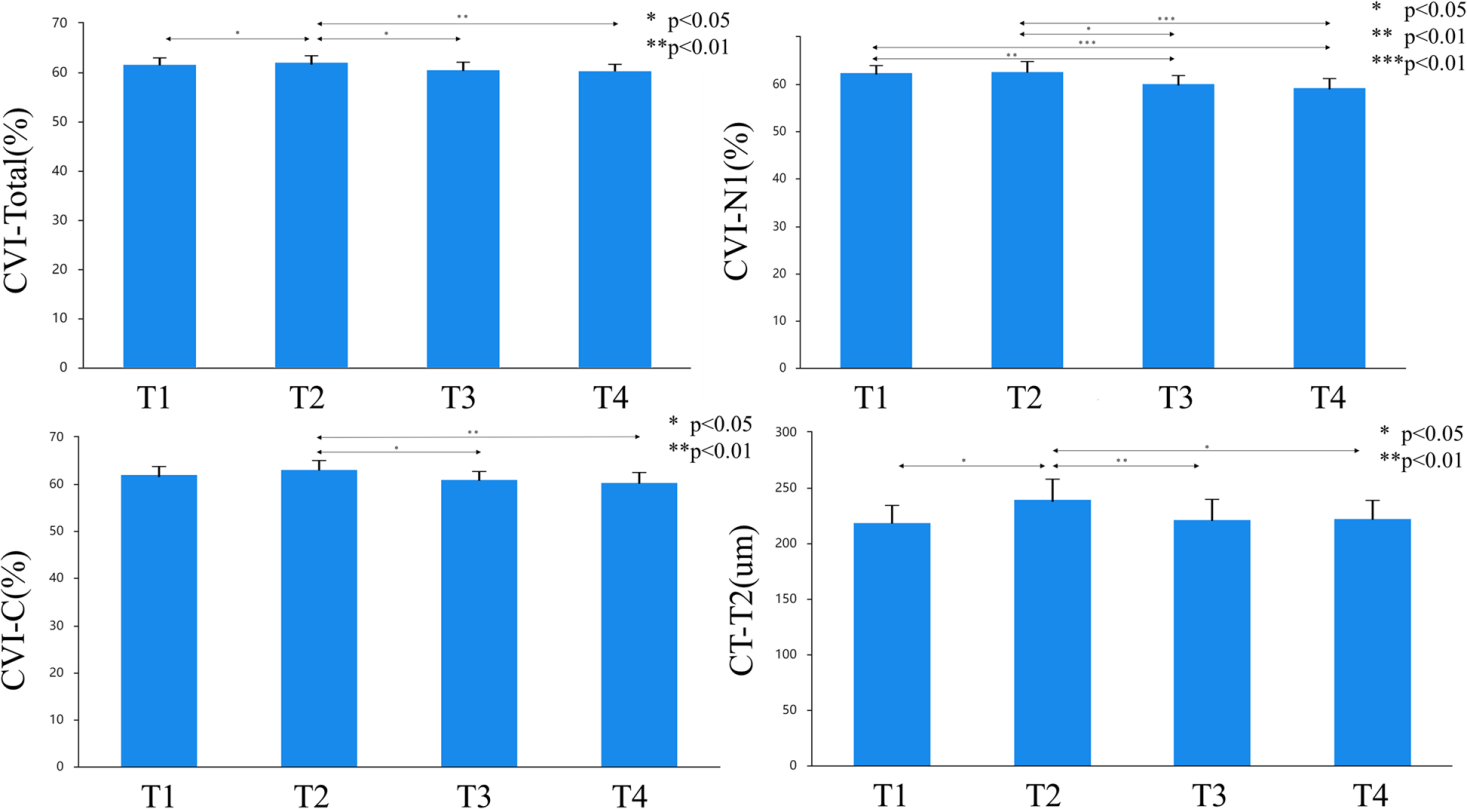
Histogram of choroidal vascularity index (CVI) in epiretinal membrane (ERM) eyes before and after surgery. T1, 1 day before surgery; T2, 1 week after surgery; T3, 1 month after surgery; T4, 3 months after surgery

A significant difference was found in the total, N1, and central CVI and CT at T2 in eyes with ERM after surgery (*p* < 0.05, Table 3). Compared to preoperative values, the CVI in the total macula increased at one week after surgery (62.14 ± 5.02%). Furthermore, the total CVI decreased at one month (60.76 ± 4.97%) and three months (60.4 ± 4.83%) postoperatively. The CVI in N1 decreased at 1 and 3 months (60.31 ± 6.15% and 59.46 ± 7.22%, respectively) postoperatively compared to that preoperatively (62.51 ± 5.93%) and at 1 week (62.82 ± 7.74%) postoperatively. In the central macula, CVI decreased at 1 month (61.18 ± 6.37%) and 3 months (60.45 ± 8.20%) after surgery compared to that at one week after surgery (63.19 ± 7.09%). CT in T2 increased at 1 week (239.65 ± 72.98um) after surgery compared with that preoperatively (218.74 ± 62.61 μm), and decreased at 1 month (222.15 ± 71.91 μm) and 3 months (222.33 ± 65.72 μm) postoperatively compared to 1 week postoperatively (Table 3) (Fig. 2).

**Table 3:** CVI and CT Variation after surgery

	1 Day Before Surgery	1 Week After Surgery	1 Month After Surgery	3 Months After Surgery	*P* Value	Post-Hoc
CVI (%)						
Total	61.70 ± 5.17	62.14 ± 5.02	60.76 ± 4.97	60.4 ± 4.83	< 0.05	^ade^
N2	58.74 ± 7.65	58.47 ± 7.95	58.26 ± 7.68	57.68 ± 7.04	0.681	–
N1	62.51 ± 5.93	62.82 ± 7.74	60.31 ± 6.15	59.46 ± 7.22	< 0.001	^bcde^
C	62.11 ± 6.84	63.19 ± 7.09	61.18 ± 6.37	60.45 ± 8.20	< 0.05	^de^
T1	62.32 ± 7.71	62.80 ± 6.4	61.27 ± 6.54	61.68 ± 6.52	0.28	–
T2	62.24 ± 7.97	63.14 ± 6.99	62.24 ± 6.95	62.54 ± 6.95	0.74	–
CT (μm)						
Total	211.41 ± 64.04	223.79 ± 72.14	216.17 ± 71.10	217.97 ± 69.25	0.13	–
N2	178.88 ± 73.08	190.15 ± 83.85	184.12 ± 81.95	183.35 ± 82.72	0.497	–
N1	209.11 ± 75.69	220.36 ± 82.51	218.07 ± 80.80	225.25 ± 92.54	0.147	–
C	226.45 ± 80.14	232.37 ± 83.75	231.13 ± 82.74	233.52 ± 82.23	0.719	–
T1	236.43 ± 75.53	245.30 ± 81.85	238.38 ± 82.08	240.54 ± 75.49	0.561	–
T2	218.74 ± 62.61	239.65 ± 72.98	222.15 ± 71.91	222.33 ± 65.72	< 0.01	^ade^

*ERM* epiretinal membrane, *CVI* choroidal vascularity index, *CT* choroidal thickness

^a^ Significant differences between 1 day before surgery and 1 week after surgery

^b^ Significant differences between 1 day before surgery and 1 month after surgery

^c^ Significant differences between 1 day before surgery and 3 months after surgery

^d^ Significant differences between 1 week after surgery and 1 month after surgery

^e^ Significant differences between 1 week after surgery and 3 months after surgery

## Discussion

In this study, we observed a higher CVI 1 week after surgery and a lower CVI at 1 month and 3 months postoperatively in eyes with ERM. The change was pronounced in the nasal and central regions. Further, we observed that CT increased after surgery and subsequently declined in eyes with ERM. Better visual acuity was observed postoperatively. Moreover, the preoperative CVI was higher in eyes with ERM than in fellow eyes.

With the development of the SD OCT technology, choroidal structures can be visualized in vivo [16]. EDI-OCT improves the visibility of the choroid–sclera interface and enables quantitative CT evaluation. More recently, CVI, a new quantitative imaging biomarker, has been shown to be a viable tool for establishing an early diagnosis, monitoring disease progression, and evaluating post-operative recovery [7]. CVI has been found to change in many ocular diseases [17–20] and altered after ophthalmic surgeries such as phacoemulsification [21, 22] and scleral buckling [23, 24]. In the current study, compared to CT, CVI had a higher sensitivity after ERM surgery. In addition, we calculated it using a self-developed semiautomatic software program with high repeatability [25].

Previous studies have evaluated CVI changes in patients with ERM after surgery. Rizzo et al. found that the CVI of eyes with ERM decreased at 1 and 3 months after vitrectomy [26]. Chun et al. reported a long-term decrease in CVI in the PPV-alone group and a short-term increase in the PPV combined with cataract surgery group [27]. However, these studies analyzed CVI changes over a longer postoperative period. Short-term complications after vitrectomy, such as hypotony and intraoperative retinal tears, have been reported [9, 28]. Therefore, short-term postoperative changes are also worthy of attention. Moreover, we measured CVI and CT in subdivisions according to the Early Treatment Diabetic Retinopathy Study rings using a semi-automatic software, which can provide choroidal data in specific regions.

A significantly higher CVI 1 week after surgery and a lower CVI at 1 month and 3 months postoperatively were the main OCT findings in this study. We hypothesized that this may be due to several mechanisms. First, early studies have reported that phacoemulsification can cause choroidal thickening due to surgical trauma-induced inflammation [21, 22]. After vitrectomy combined with phacoemulsification, the choroidal vascular area grows. This is possibly due to a disturbance in the blood–aqueous barrier, which enables inflammatory mediators from the aqueous to cross the vitreous and enter the choroid, causing structural alterations [7]. This could explain the increased CVI and CT at the early stage in this study. Second, vitreomacular tension may stretch the RPE and stimulate vascular endothelial growth factor release, resulting in an increased choroidal vascularity [29]. Vitrectomy eliminates vitreomacular traction, causing vascular endothelial growth factor levels to decrease. This explains why eyes with ERM had a greater CVI preoperatively and a lower CVI one month and three months postoperatively in our study. Third, the choroidal structure may change due to alterations in the retina, with the central and nasal choroids displaying higher effects than other regions. Hibi et al. reported a significant reduction in inner and middle retinal layers after ERM surgery [30]. Further, Park et al. reported progressive thickening of the nasal inner nuclear layer after ERM surgery [31]. These retinal changes might explain the pronounced central and N1 changes in the postoperative CVI. In addition, prominent changes in the nasal region of the macula may be related to the special anatomy of the retina, such as the papillomacular bundle. Different alignments of the papillomacular bundle between the nasal and temporal sides may lead to a more sensitive CVI on the nasal side. Although we cannot confirm that changes in retinal structure lead to changes in CVI, our results nonetheless suggest that the postoperative CVI of eyes with ERM, especially CVI in the central and nasal regions, may be a useful and objective method of assessing changes in choroidal vascular structure during the postoperative period. A sensitive technique for evaluating the choroidal vasculature may help clinicians to improve monitoring during follow-up.

**Fig. 3:**
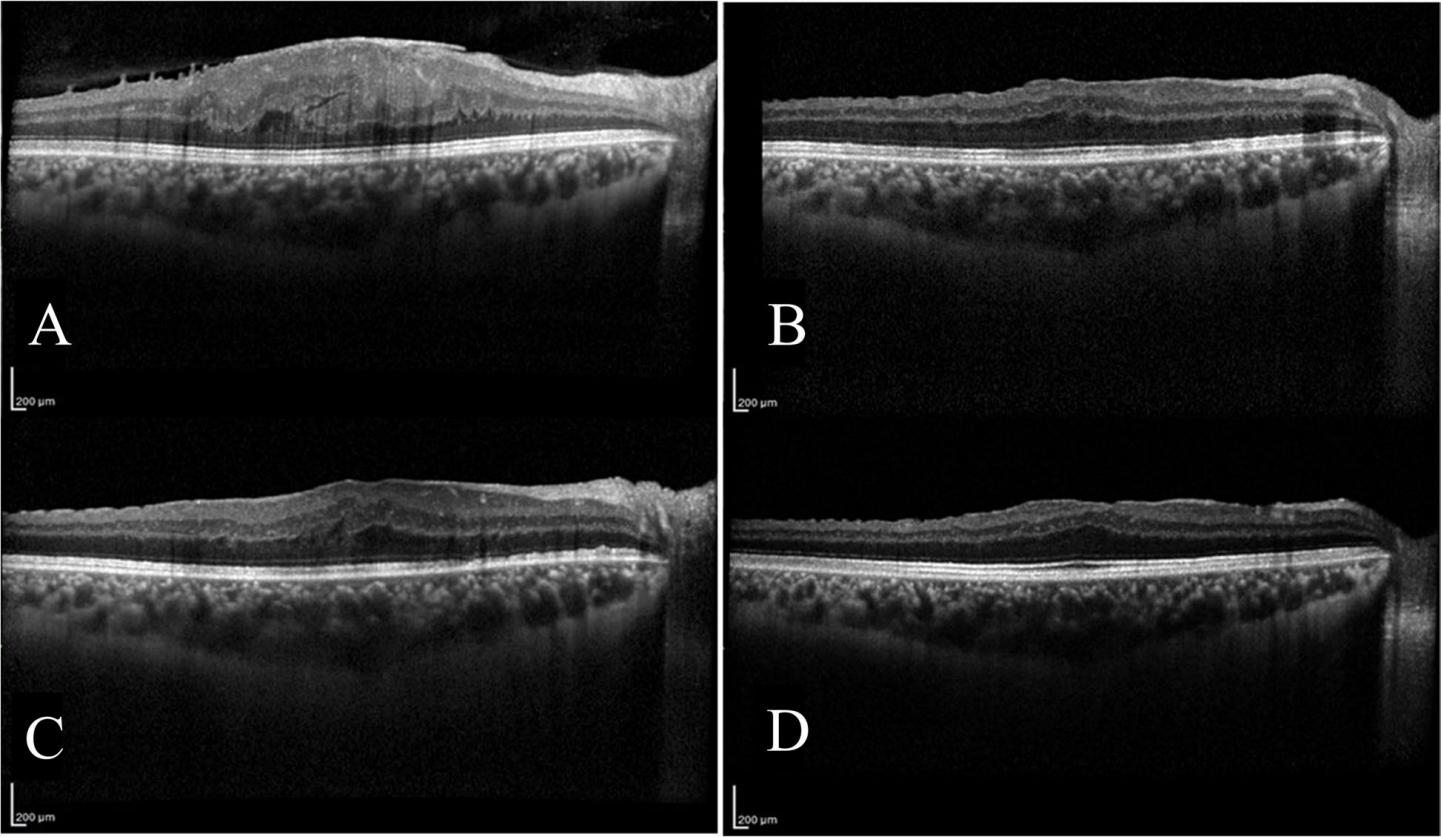
OCT B-scans of an eye with epiretinal membrane for preoperative period and all postoperative periods. **A**, 1 day before surgery; **B**, 1 week after surgery; **C**, 1 month after surgery; **D**, 3 months after surgery

This study had several limitations. First, the patient cohort was not sufficiently large. Second, this was a retrospective study, which carries a risk of sample bias. Third, we did not use swept-source OCT to obtain B-scan images with higher penetration. However, Agrawal et al. demonstrated that CVI measurements obtained using SS-OCT and SD-OCT concur with each other [16]. Fourth, because the location of anatomic check points (vessels and fovea) may change after surgery, to scan the same location of choroidal area during follow-up is difficult. This could be one of the reasons for the conflicting results in this literature regarding macular surgery and CVI. However, we used the follow up mode in OCT scanning, which could minimize this scanning deviation (Fig. 3). Moreover, we excluded subjects with obvious change in choroidal area during follow-up. Fifth, in this study, only half of the ERM patients had the angiographic examination such as fluorescein angiography or indocyanine angiography to exclude abnormal retinal or choroidal circulation. And none was reported to have obvious vasculature disruption in retina and choroid. Sixth, we used paired t-tests to compare CVI and CT between eyes with ERM and fellow eyes. CVI comparison with the fellow eye reduce interpersonal CVI difference, but an intrapersonal CVI difference still exists.

In conclusion, CVI of eyes with ERM increased in the early postoperative period and then decreased, especially in the central and nasal regions. Further prospective studies with larger sample sizes are required to confirm the effect of vitrectomy combined with cataract surgery on choroidal structure.

## Conclusions

In this study, we investigate short-term choroidal structural and vascular changes after epiretinal membrane (ERM) surgery using enhanced depth imaging optical coherence tomography. We found a significantly higher CVI 1 week after surgery and a lower CVI at 1 month and 3 months postoperatively. The change was pronounced in the nasal and central regions. This may be related to postoperative inflammation and the release of macular traction. Therefore, CVI in the central and nasal regions, may be a useful and objective method of assessing choroidal blood-flow changes during the postoperative period. A sensitive technique for evaluating the choroidal vasculature may help clinicians to assess choroidal structural changes accompanying ERM and postoperative period.

## Data Availability

The data that support the findings of this study are available from the corresponding author upon reasonable request.
